# Uncommon Presentation of a Symphyseal and Bilateral Mandibular Body Fracture From a Gunshot Injury: A Case Report and Literature Review

**DOI:** 10.7759/cureus.81052

**Published:** 2025-03-23

**Authors:** David A Cruz Walma, Boyu Ma, Somsak Sittitavornwong

**Affiliations:** 1 Department of Orthodontics, Harvard School of Dental Medicine, Boston, USA; 2 Department of Oral and Maxillofacial Surgery, University of Alabama at Birmingham, Birmingham, USA

**Keywords:** dental malocclusion, major trauma, mandibular fracture, mandibular reconstruction, oral reconstruction

## Abstract

Mandibular fractures are one of the most common types of facial fractures and often result from trauma to the head and neck region. Understanding the mechanism-based factors resulting in different patterns of mandibular injury is important for their surgical management. This study reports a unique mandibular fracture whereby the symphysis and bilateral mandibular bodies were fractured following a gunshot wound to the maxillofacial region. A literature review of case reports on bilateral mandibular fractures highlights the uniqueness of the presented case and supplements the text as the initial management, treatment, and prognosis of the case are discussed.

## Introduction

Mandibular fractures are among the second most common type of facial fractures, accounting for 27.4% of all facial fractures as mandible fractures [1-3]. Due to the prominence of the mandibular symphysis, the mandible is highly susceptible to blunt traumatic injuries resulting from motor vehicle accidents, falls, assaults, and other mechanisms [1]. Mandibular fractures are classified based on their impact on bone (e.g., direct fracture or indirect fracture), displacement of fracture fragments (e.g., vertically or horizontally favorable/unfavorable), and site of fracture (e.g., region of the condylar process, coronoid process, ramus, angle, body, alveolar process, or symphysis of the mandible) [4].

Retrospective epidemiologic studies analyzing thousands of mandibular fractures have shown that approximately half occur at a single site, while the other half involve multiple sites. Two-site fractures account for roughly 34% of cases, and three-site fractures account for approximately 5% [4]. Although incidence rates vary among studies, most agree that the mandibular angle, symphysis, and condyle are the most frequently fractured anatomic locations following trauma [5-8].

Common mechanisms of mandibular injury include assaults, motor vehicle collisions, and falls, while a significantly smaller percentage result from firearms and sports-related injuries [5,8]. Firearm-related injuries account for approximately 3.1% of mandible fractures. [9] Several large epidemiologic studies have examined the association between specific fracture sites in cases of multiple-site mandibular fracture [4]. Bilateral mandibular fractures have been described to account for 43.9% of cases [2]. These studies have demonstrated a strong correlation between symphysis fractures and condyle fractures as force applied to the mandibular symphysis is transmitted directly to the condylar region [4]. However, these studies do not report if the fractures are bilateral, nor do they establish a strong link between symphysis fractures and mandibular body fractures, particularly bilateral body fractures. Furthermore, Morris et al. [5] retrospectively analyzed 4,143 mandibular fractures and concluded that a strong correlation exists between symphysis and condyle fractures.

Firearm-mediated mandibular fractures are relatively uncommon, and bilateral mandibular body fractures are rare as well [5,8]. We present a case of symphysis and bilateral mandibular body fractures following a gunshot wound to the face. Due to the rarity of this fracture pattern, we conducted a literature review of reported cases of bilateral mandibular fractures from 1970 to the present. To our knowledge, this is the first reported case of symphysis fracture with concomitant bilateral mandibular body fractures.

## Case presentation

**Figure 1:**
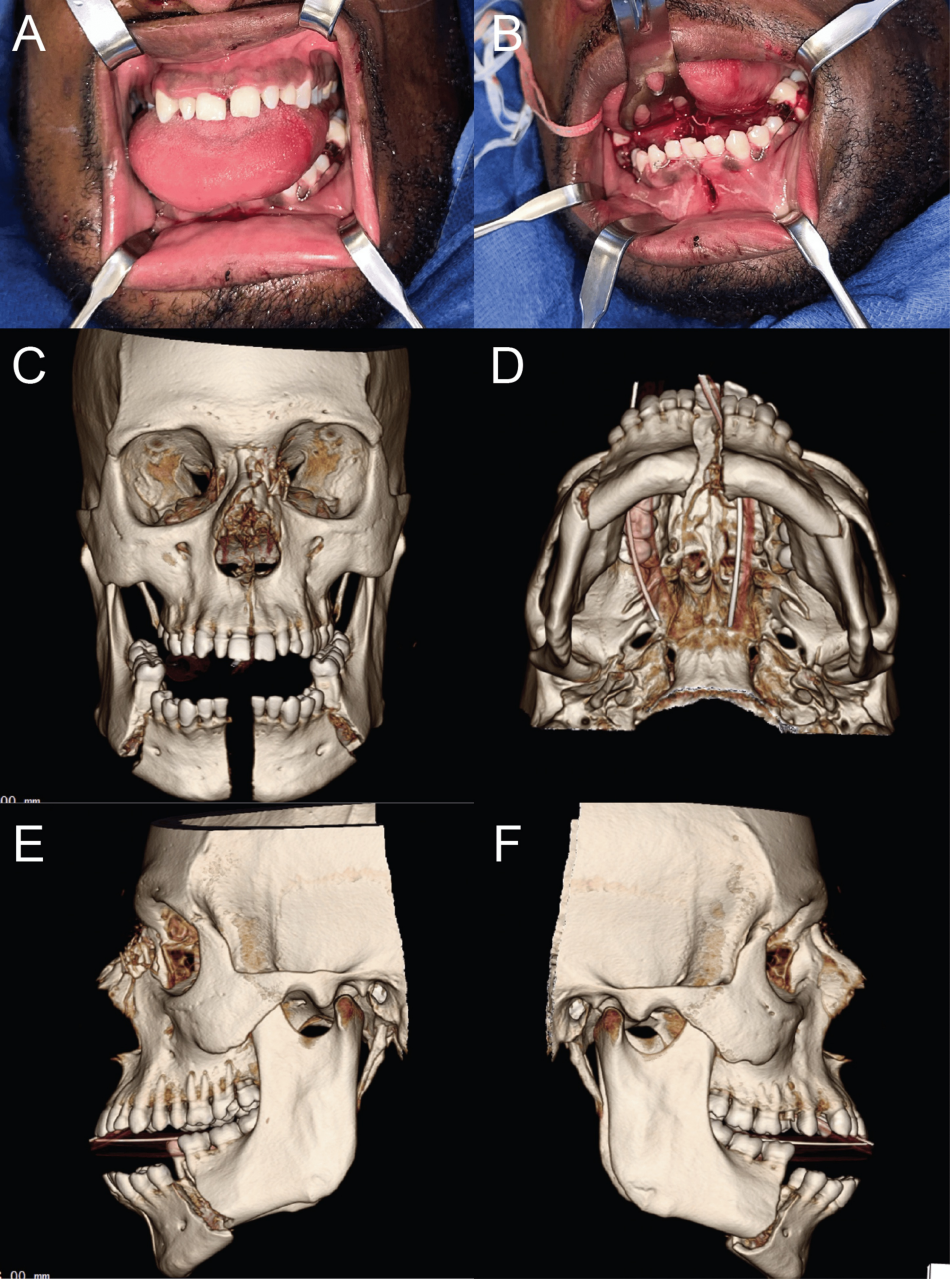
Preoperative presentation and 3D reconstruction of maxillofacial computed tomography scan. (A,B) Clinical photographs showing the fracture sites from two different angles. 3D reconstructions of full-face computed tomography scans showing the mandibular fractures from the (C) frontal, (D) coronal, (E) left sagittal, and (F) right sagittal views

A 35-year-old African American man presented to the trauma bay at the University of Alabama Hospital following a self-inflicted gunshot wound to the chin. The patient was admitted to the trauma intensive care unit service with multiple injuries, including an obvious anterior mandibular fracture with misalignment of lower teeth, tongue laceration, and gunshot wound to the right lateral chin (Figures 1A, 1B). As the patient was intubated and sedated before arrival, his past medical history was unknown. No relevant past interventions of the head were known, and no previous injuries or incisions were seen outside of the primary injury. Clinical examination supplemented by maxillofacial computed tomography (CT) revealed 4 x 4 cm entry and exit wounds at the submental and forehead areas, respectively. The segments were displaced by approximately 4 mm at each body fracture and 8 mm at the symphysis fracture. Imaging demonstrated open, completely comminuted segmental mandibular fractures between central incisors as well as bilateral molars and premolars, and complex comminuted maxillofacial fractures involving the hard palate, maxillary alveolar ridge, and left nasal orbital ethmoid complex (Figures 1C-1F). Based on the injury pattern, it was determined that the bullet projectile entered from the submental area and passed through the mandible, floor of the mouth, and palate, and exited out of the nasal bone. On the day of admission, informed consent was obtained, and laceration repair of the submental and forehead areas was performed using standard sterile techniques. Iodoform gauze was packed in the submental and forehead wounds after irrigation with sterile saline. Bridle wires, 26 gauge, were placed around teeth 19 and 21, 23 and 26, and 28 and 30 to stabilize the mandibular segments until surgery (Figures 1A, 1B).

**Figure 2:**
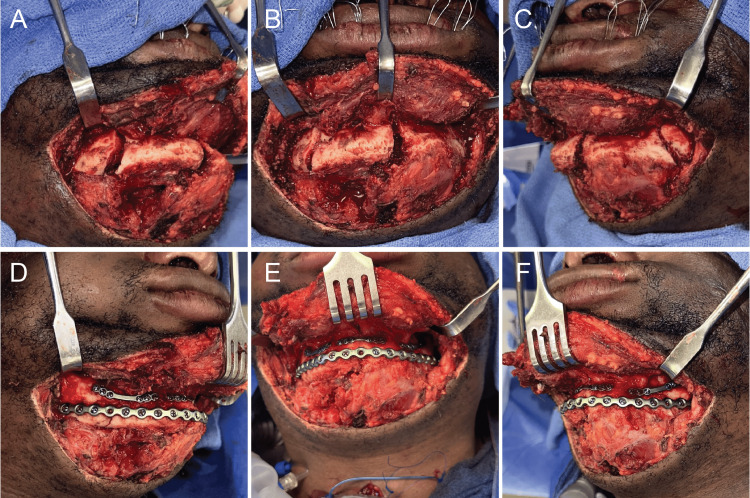
Surgical views of mandibular fracture pre- and postfixation. Clinical photographs with soft tissue reflected showing the three mandibular fracture sites prior to fixation in the (A) right, (B) front, and (C) left orientations. Clinical photographs with soft tissue reflected showing the three mandibular fracture sites after fixation in the (D) right, (E) front, and (F) left orientations

**Figure 3:**
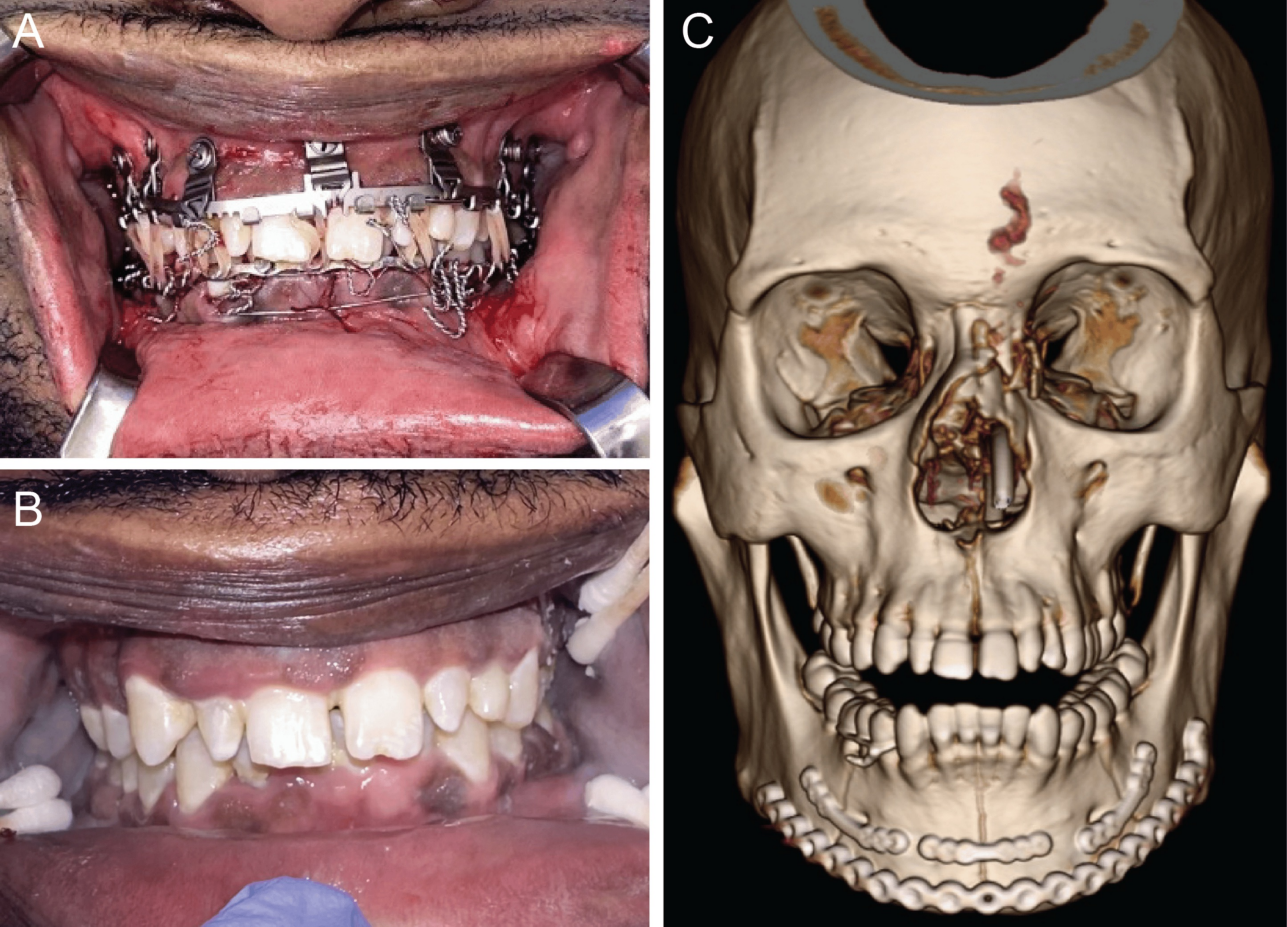
Postoperative occlusion with intermaxillary fixation, 3D reconstruction of maxillofacial computed tomography scan, and occlusion at two-week follow-up examination. (A) Clinical photograph showing intermaxillary fixation with 26-gauge wires around hybrid arch bars. (B) Clinical photograph of patient in occlusion following removal of intermaxillary fixation. (C) 3D reconstruction of full-face computer tomography scan after removal of intermaxillary fixation showing successful reduction and fixation of the mandibular fracture sites

One day later, the surgery was performed under general anesthesia, with a tracheostomy established for airway management. Hybrid arch bars were fixed to the maxilla with screws, ensuring careful attention to the maxillary tooth roots. Traditional Erich arch bars were placed around the mandible using 24- and 26-gauge wires to help reduce the fractures and act as a tension band. Then, intermaxillary fixation was achieved using 26-gauge wires around hybrid arch bars to align the occlusal plane and reduce the fractures both functionally and anatomically. A transcervical approach, which allowed easier access to reduce the fractures and direct view of all three sites, was used to expose the fractured mandibular segments, revealing comminuted fractures of the right mandibular body, left mandibular body, and mandibular symphysis (Figures 2A-2C). To ensure proper alignment of the occlusal plane, the right mandibular body fracture was reduced and fixated with a 1.0-mm tension band, and then, the symphysis and left mandibular body fractures were sequentially reduced and fixated in a similar manner (Figures 2D-2F). At the inferior border of the mandibular, a compression band was applied, and a 2.5-mm titanium reconstruction plate (KLS Martin, Jacksonville, FL) encompassing the three fracture segments was placed with screws (Figures 2D-2F). Intermaxillary fixation was removed, and a stable and repeatable occlusion was verified (Figure 3). After addressing the mandibular fractures, a closed reduction of the lateral nasal bone fractures was performed, and a nasal dressing was applied.

**Figure 4:**
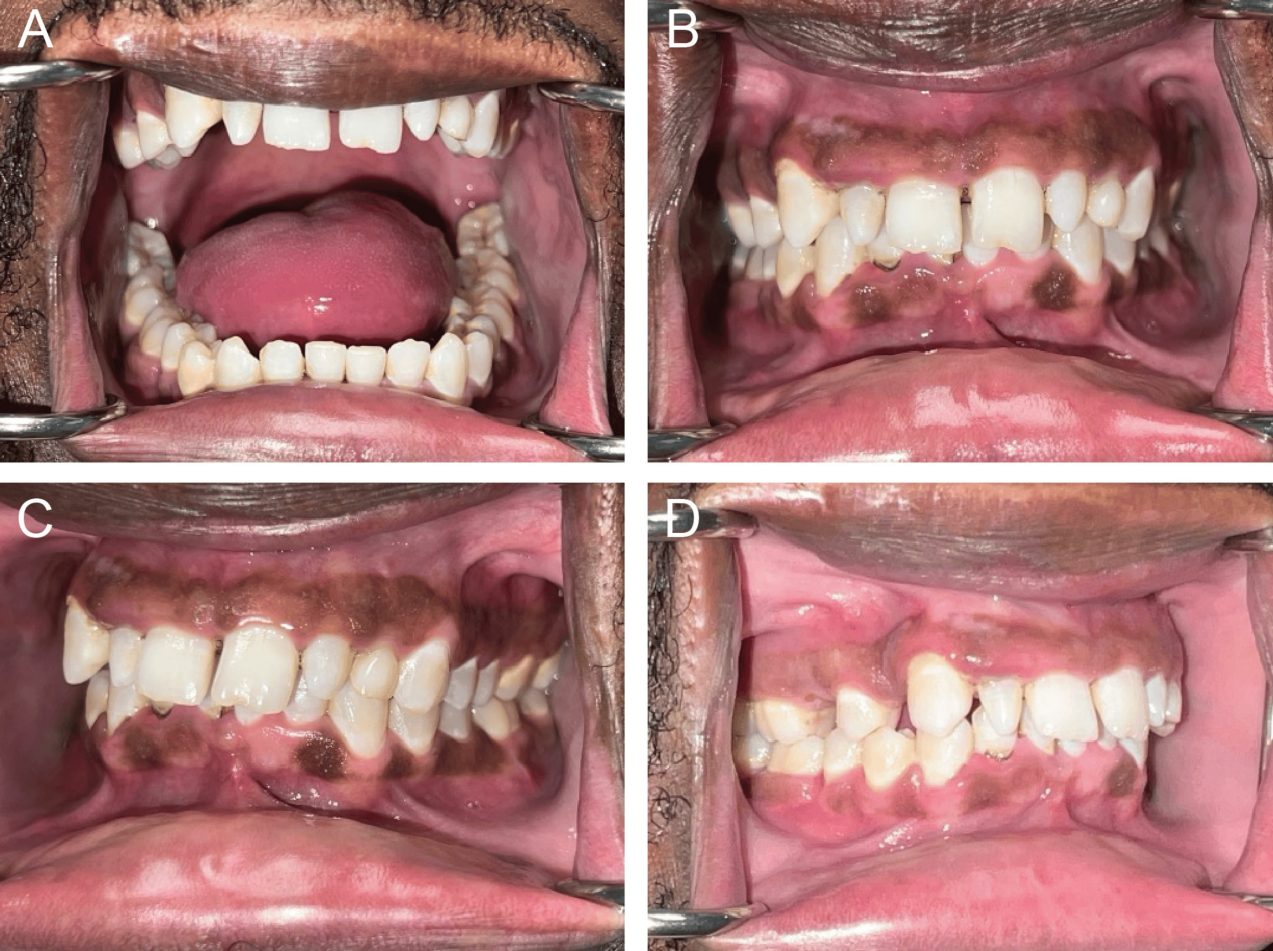
Occlusion at six-week postoperative examination. Clinical photographs taken at a six-week postoperation examination showing the patient has satisfactory mouth opening (A) alongside a stable occlusion with images depicting the patient occluding in maximum intercuspation from the (B) front, (C) right, and (D) left views

Postoperative CT maxillofacial scan demonstrated a satisfactory reduction of the fractures and contour of the reconstruction plate (Figure 3B). Clinically, the patient had a stable and repeatable occlusion with some bilateral hypoesthesia of the mandibular division of the trigeminal nerve. The occlusion was stable and repeatable. Two weeks after surgery, the patient was extubated, and the occlusion remained stable and repeatable (Figure 3C). The patient had lost over 10 kg. The patient continued to have V3 hypoesthesia and notable lower facial swelling that was improving. At the most recent follow-up examination six weeks after surgery, the patient was healing satisfactorily, no clinically significant facial asymmetries were observed, and the patient’s occlusion was normal with a full range of jaw movements devoid of pain or sensory disturbances (Figure 4). The patient was on a soft, nonchewing diet at this visit. Physical examination showed a stable and repeatable occlusion, improved maximal incisal opening, and no pain with jaw movements. He reported improved speech. The patient had also gained back 5 kg. The patient was then advanced to a regular chewing diet after six weeks of healing to allow the bone to fully strengthen. His hypoesthesia had improved bilaterally with a return of pinprick sensation and sense of direction. Overall, the patient had no adverse events in the postoperative period (Table 1).

**Table 1:** Timeline of events from initial injury to surgery and postoperative period CT: computed tomography

Timeline	Clinical finding
Initial injury	Self-inflicted gunshot wound to the chin causing completely comminuted segmental mandibular fractures of the bilateral body and symphysis of the mandible
Operation	Erich arch bar used to reduce the occlusion. A transcervical approach was used to expose the fractures. Then, open reduction internal fixation of the fracture sites was completed with titanium plates (KLS Martin)
Immediate postop	Satisfactory reduction of fractures and with stable and repeatable occlusion. CT maxillofacial was done to confirm this
Two-week postop	10-kg weight loss with stable and repeatable occlusion. Decreased swelling. V3 present hypoesthesia
Six-week postop	5-kg weight gain with stable and repeatable occlusion. The patient is okay to advance diet. Improvement in speech. Improved V3 hypoesthesia. No adverse events during the postoperative period

## Discussion

The mandible is one of the most frequently injured bones of the maxillofacial region. Several multicenter retrospective epidemiologic studies have explored how various factors relate to traumatic injuries of the mandible [5-9]. Most of these studies have concluded similar results, with minor deviations between cohorts. The most common causes of mandibular fractures include motor vehicle accidents, physical assault, and falls [5,6,8]. The most common sites of mandibular fracture, in descending order of prevalence, include the mandibular angle, symphysis, condylar/subcondylar, and body [5]. Approximately half of mandibular fractures exist as single-site fractures, with the other half of mandibular fracture injuries involving multiple fracture sites [5]. Morris et al. investigate site association between different locations of mandibular fractures by noting a fracture at one site and then determining the incidence of fracture at a second site [5]. From their analysis, symphysis fractures most commonly presented with a second fracture at the mandibular angle or condylar/subcondylar region [5]. Very few symphysis fractures (only 7.4% of all symphysis fractures with fracture at a second site) were associated with a mandibular body fracture [5]. Unfortunately, these retrospective studies did not specify if the mandibular body fractures associated with symphysis fractures were unilateral or bilateral.

A comprehensive literature review was conducted utilizing PubMed and Google Scholar to identify scientific articles detailing cases of simultaneous bilateral mandible fractures. The search employed keywords such as “bilateral mandible fracture,” "bilateral mandibular condylar fracture", "bilateral mandibular angle fracture," "bilateral mandibular body fracture," and "bilateral mandibular coronoid fracture." Inclusion criteria were restricted to studies published in English that described types of bilateral mandible fractures. The search strategy identified 60 reports of bilateral mandibular fractures reported between 1970 and the present day (Table 2). Similar trends to those reported in retrospective reviews were observed in these case reports, with motor vehicle accidents, physical assault, and falls being the most common causes of mandibular fracture. Other reported causes included kicks from horses, falls from bicycles, pathologic fractures secondary to disease, fractures associated with the placement of endosseous dental implants, and injury during physical convulsions. Notably, no cases in the literature reported a symphysis with bilateral mandibular body fracture, and no cases reported bilateral mandibular fractures associated with a gunshot wound (Table 2), further emphasizing the uniqueness of the presented case. Morris et al. reported mandibular fractures caused by firearms commonly resulted in mandibular body fractures [5]. However, they explained that firearm injuries typically resulted in unpredictable “nonanatomic patterns of injury” dependent on the random path of the projectile as it ricochets off tissues, rather than the biomechanical properties of the mandible [5]. In the presented case, the bullet projectile entered from the submental area and passed through the mandible, floor of the mouth, palate, naso-orbitoethmoidal region, and the soft tissue of the left medial frontal region. The patient likely then fell and struck his chin, resulting in the observed mandibular fractures. Falls rarely result in symphysis fractures with concomitant bilateral mandibular body fractures (no reported cases located) and instead typically result in bilateral condylar fractures. In our reported case, the projectile passed near the mandibular symphysis and lacerated several muscles and connective tissues that insert into the anterior mandible. The ballistic trauma could have compromised the structural integrity of the anterior mandible and predisposed the region to fracture. As a result, instead of forces from a strike to the chin (secondary to a fall) being transmitted to the condyles (with subsequent condyle fracture), the anterior mandible was unable to withstand the forces associated with a fall and direct strike to the chin, thus leading to the observed symphysis and mandibular bodies fractures.

**Table 2:** Analysis of bilateral mandibular fracture cases reported in the English literature between 1970 and 2024, indexed to Google Scholar, PubMed, and Scopus CT: computed tomography; POD: postoperative day; TMJ: temporomandibular joint; BSSO: bilateral sagittal split osteotomy; PCC: prothrombin complex concentrate

Author/year	Number of mandibular fracture sites	Gender/age at the time of presentation (year)	Dentition	Etiology	Symptoms	Fracture location(s) and other image findings	Management	Complications/outcome
Bilateral condylar fractures								
Sanger and Greer [10]	3	M/74 years	Edentulous	Motor vehicle accident 16 years before	None reported	Bilateral condylar fractures and left mandibular body fracture were diagnosed at the time of the accident (16 years prior)	No treatment reported	The patient was asymptomatic for temporomandibular joint problems and had a normal range of motion of the mandible at 16 years postinjury
Miller and McDonald [11]	2	M/9 years	Dentate, mixed	Fall from bicycle	Open bite deformity, limited mouth opening (16 mm), mandibular deviation to the left, bilateral pain in the region of the temporomandibular joint	Initial panoramic film and mandibular radiographs could not detect a fracture. Four weeks later, a CT scan revealed medially displaced bilateral condylar fractures with comminution of the left condyle	Nonsurgical therapy. During the first four weeks without a diagnosis of condylar fracture, only physical therapy was performed. Upon diagnosis of condylar fractures, maxillomandibular fixation was applied using Erick arch bars (Novo Surgical, Westmont, IL) for two months, followed by training elastics to allow limited mandible function for another two months	At the six-month postinjury exam, occlusion was stable and reproducible. At the 10-month postinjury exam, a CT scan showed complete remodeling of both condyles
Bavitz and Collicott [12]	3	M/71 years	Dentate, permanent	Struck by a motor vehicle while walking	Not reported	Bilateral dislocated fractures of right and left condylar processes, compound right parasymphyseal fracture	Open reduction and rigid internal fixation of right parasymphyseal fracture. Initially, no treatment was provided for bilateral subcondylar fractures. However, due to complications, in a follow-up procedure 10 days postop, closed reduction of condyle fractures and intermaxillary fixation was provided with a Gunning splint (Alibaba, Hangzhou, China)	During a follow-up procedure 10 days postop, the patient went into respiratory distress that was hypothesized to be caused by a combination of the preoperative sedatives and untreated subcondylar fractures. Following a closed reduction of condyles at a follow-up appointment, no further complications were reported
Jouffre et al. [13]	2	F/69 years	Edentulous	Fall	Preauricular regions tender to palpation	Bilateral high condyle fracture with dislocation and left temporal fracture	Nonsurgical treatment. Physical therapy encouraging early active motion of the mandible without elastic traction for six weeks	No modification to occlusal relationship, trismus, pain, or instability. No functional deficits. No resorption of fractured condylar heads
Luz and Chilvarquer [14]	2	M/11 years	Dentate, mixed	Fall	Bilateral preauricular swelling	Bilateral fracture of condylar necks with medial and anterior dislocation of proximal fragments	Nonsurgical treatment. Conservative management with short-term rigid intermaxillary fixation (seven days) followed by elastic intermaxillary fixation (seven days) with physical therapy, and finally a removable space-maintaining partial denture	At a 35-month postinjury follow-up exam, the patient was asymptomatic, maximal jaw opening was 46 mm, and radiographic changes showed a return to the normal shape and position of the condyles
Matteini and Belli [15]	3	M/31 years	Dentate, permanent	Motor vehicle accident	Difficulty breathing, facial swelling, open bite	Bilateral subcondylar fractures and parasymphyseal fracture	Reduction and rigid internal fixation of parasymphyseal fracture. No surgery was performed on condyles. Intermaxillary fixation for 10 days	No surgical complications. A severe limitation in jaw movements (maximum opening of 9 mm) developed. Bilateral ankylosis of condyles was noted six months after surgery. Ankylosis was treated surgically with the removal of ankylotic blocks and condylar stumps
Dang [16]	2	F/19 years	Dentate	Fall	Difficulty opening	Bilateral mandibular condyle	Open reduction internal fixation	No complications reported
Watanabe et al. [17]	2	M/51 years	Dentate, permanent	Fall	Not reported	Bilateral condylar neck fractures	Internal distraction devices are placed bilaterally and used to slowly reduce the dislocated condylar heads. Three days after surgery, continuous intermaxillary fixation using elastics was applied for 14 days and thereafter continued with only night-time use for six weeks. The distraction device was removed after 16 weeks. Physical therapy performed throughout the treatment	No surgical complications were reported. Six months after the removal of the distraction device, maximum mouth opening was reported to be 46 mm. One year after surgery, a computed tomography scan showed remodeling and restoration of bilateral condylar processes
Medina [18]	2	F/7 years	Dentate, mixed	Fall from second-floor balcony	Limited mouth opening, facial asymmetry, mandibular deviation to the left during opening	Bilateral condylar fracture with medial displacement, vertical fracture of the mandibular body	Extraction of vertically fractured primary molars, soft diet, and physical therapy for seven days. Functional appliance therapy using a modified Klammt elastic elevator (GerDentUSA Inc., Oakland Gardens, NY) that advanced and vertically displaced the mandible to allow condylar remodeling. The patient then received comprehensive orthodontic therapy	At the six-year follow-up exam, remodeling of condyles was observed with “good to excellent anatomy,” normal occlusion and jaw movements observed
Lee and Kim [19]	3	M/47 years	Dentate, permanent	Not reported	Anterior open bite, crossbite	Bilateral condylar fractures (left subcondylar fracture, right condylar neck fracture), parasymphyseal fracture, palatal fracture	Internal reduction and fixation of parasymphyseal and palatal fractures. Intraoral manual reduction and extraoral fixation of condylar fractures using distraction osteogenesis devices, as well as intermaxillary fixation using arch bars and elastic bands for three weeks. The right side condylar fracture had to be reduced a second time. External condylar fixation was removed after five weeks	No surgical complications were reported. At the three-week postoperative exam, the right side condylar fracture had to be reduced a second time. At a five-week postoperative exam, open bite worsened but “improved spontaneously.” At the three-month postoperative exam, mouth opening was 38 mm, and “no clinically significant malocclusion or facial asymmetry was observed”
Dölekoğlu et al. [20]	3	M/30 years	Dentate, permanent	Fall	Limited mouth opening	Bilateral condylar fracture, alveolar fracture near left mandibular incisor region, alveolar fracture near upper right molars	Referred to a state hospital for surgery; no surgical treatment was reported	No follow-up exams reported
Chakraborty [21]	3	M/28 years	Dentate	Blunt trauma	Anterior open bite, restricted mouth opening	Mandibular parasymphysis​​​​​ fracture and bilateral mandibular condyle	Open reduction internal fixation	No reported complications
	2	M/13 years	Dentate	Fall	Difficulty opening	Bilateral mandibular condyle	Open reduction internal fixation	No reported complications
	2	M/27 years	Dentate	Fall	Difficulty opening	Bilateral mandibular condyle	Open reduction internal fixation	No reported complications
	3	M/32 years	Dentate	Blunt trauma	Difficulty opening	Mandibular symphysis, bilateral mandibular condyle	Open reduction internal fixation	No reported complications
Park et al. [22]	3	M/22 years	Dentate, permanent	Fall	Initial presentation: not reported. Secondary presentation prior to additional therapy: limited mouth opening (25 mm) with posterior and left lateral displacement of mandible, ankylosed condyle, severe class II skeletal relationship, several missing teeth	Initial presentation: bilateral condylar fracture, parasymphyseal fracture. Secondary presentation: ankylosed left condyle	Initial surgery: open reduction and fixation of parasymphyseal fracture, closed reduction of bilateral condylar fractures. Subsequent surgical and prosthetic procedures: full mouth prosthetic rehabilitation including ceramic veneers, bilateral sagittal split ramus osteotomy and simultaneous placement of nine dental implants, maxillomandibular fixation for three weeks, second stage surgeries and conventional workflows to restore implants	No reported surgical complications. At the follow-up exam one year after full mouth rehabilitation, no pain, clicking/popping, nor functional abnormalities of the temporomandibular joints were present, and maximum mouth opening was 40 mm
Benaglia et al. [23]	3	M/49 years	Partially edentulous maxilla and mandible	Motor vehicle accident	Initial presentation prior to preliminary surgery: not reported. Presentation prior to secondary surgery: severely restricted mouth opening (<1 mm)	Initial presentation: bilateral condylar fracture and comminuted symphyseal fracture. Secondary presentation: bilateral ankylosed condyles	Preliminary surgery: open reduction and fixation of symphyseal fracture, exploratory surgery of condyles with preauricular access without reduction and fixation of fractures, maxillomandibular fixation for 45 days. Secondary surgery (11 years later): ankylosed bone exposed, aggressive resection of bone mass completed, leaving a gap of 15 mm between the superior border of fossa and mandible, and rigorous physical therapy performed for six months	Preliminary surgery: at a 45-day postoperative exam following removal of maxillomandibular fixation, severe restriction in mouth opening developed that did not improve with physical therapy. Upon presenting for secondary surgery 11 years later, bilateral condylar ankylosis was diagnosed with the bilateral union of the condylar heads to the temporal bones and zygomatic arch. Secondary surgery: no reported surgical complications. At the 12-month follow-up exam, a mouth opening of 35 mm was observed
Marano et al. [24]	3	M/3 years	Dentate	Fall	Swelling and bruising in the submental and floor of the mouth	Mandibular symphysis and bilateral condyle	Open reduction internal fixation	No reported complications
Gašpar et al. [25]	3	F/5 years	Dentate, primary	Fall from bicycle	Limited opening, pain upon opening, pain in preauricular region, inability to close into centric relation	Bilateral fracture of the condylar neck with dislocation of the right condylar neck, greenstick fracture of the right parasymphyseal region	Nonsurgical conservative treatment. The mandible was bimanually repositioned, standard edgewise orthodontic brackets were placed, and orthodontic rubber elastics were used for the bilateral fixation of the upper and lower first molars for two weeks. One week of active physiotherapy using elastics, then continued mouth-opening exercises for several weeks	No complications were reported. At the six-month follow-up exam, the fractures were healed, and the patient could close intro centric relation. At the two-year follow-up exam, normal mouth opening and “ideal symmetry” evidenced by clinical and radiographic evaluations
Grow et al. [26]	3	F/4 years	Dentate, primary	Motor vehicle accident	Open bite deformity, pain upon mandibular movements	Bilateral condylar fractures and symphyseal fracture with medial subluxation of both condyles and lateral displacement of bilateral body and angles	Reduction and fixation of mandibular angles via transfacial insertion of a Steinman pin through both mandibular angles passing over the base of the tongue. A tension band was placed on the lateral incisors of the mandible. Maxillomandibular fixation was applied for 14 days using intermaxillary fixation screws and circummandibular wires. Active physical therapy upon removing intermaxillary fixation. Steinman pin removed at 11 weeks after repair	No reported surgical complications. At the one-month follow-up exam, near-full excursive movements of the jaw and tongue were observed with a transfacial pin in place. At the 79-day follow-up exam (with Steinman pin removed), fracture sites healed, condylar fractures remodeled, and facial symmetry with a normal full range of jaw movements observed
Lee et al. [27]	5+	M/19 years	Dentate, permanent	Fall	Defects in mandible continuity, lack of occlusion, limited opening (less than 20 mm)	Extensive comminuted fractures of bilateral mandibular angles, bilateral condylar fractures, shattered bony fragments of the alveolus and mandibular body, avulsion and dislocation of all posterior mandibular teeth	Open reduction and internal fixation of mandibular comminuted fractures using locking reconstruction plate, mini-plates, and screws. Reduction and fixation of marginal bone segments to reconstruction plate. Reduction and fixation of small bone segments to each other and marginal bone. Closed reduction of condylar fractures. Maxillomandibular fixation for two weeks followed by rehabilitation physical therapy	No reported surgical complications. At 10-week follow-up, mouth opening improved to 30 mm (from 23 mm), and favorable bone as well as soft tissue healing was observed
Parthasarathy and Sripriya [28]	2	M/65 years	Dentate, permanent	Fall	Difficulty opening mouth	Bilateral condylar fracture with dislocation of condyles	Surgical reduction of condylar fracture-dislocations and internal fixation performed in the dental chair with regional anesthesia without sedatives	No complications reported
Hwang and Kim [29]	3	M/37 years	Dentate, permanent	Fall	Anterior open bite	Bilateral condylar fracture and left parasymphyseal fracture	Intermaxillary fixation (at posttrauma day 3) followed by extracorporeal reduction of condylar fracture (at posttrauma day 10)	By POD 9, infection of the right condyle was noted with *Streptococcus anginosus* cultured from pus draining from the surgical site on POD 18. On POD 29, the wound was debrided and drained, and the patient was discharged. On POD 39, pus discharge recurred, and condylar head was observed to have resorbed. On POD 47, the infected condylar head was resected and reconstructed with an iliac bone graft. Following condyle replacement, healing was uneventful, and incisal opening was reported to improve to 24 mm
Zhang et al. [30]	3	F/20 years	Dentate, permanent	Motor vehicle collision	Constant aching pain, malocclusion	Bilateral condylar fracture, symphyseal fracture	Open reduction internal fixation	No complications reported
Xu et al. [31]	3	M/10 years	Dentate, mixed	Motor vehicle accident	Facial pain, difficulty chewing, bilateral facial swelling, shift of mandibular facial midline, mandibular deviation upon opening and closure, limited mouth opening	Vertical fracture of left condylar head, displaced fracture of right condylar neck, mandibular symphysis fracture	Nonsurgical, conservative treatment using standard edgewise orthodontic brackets and intermaxillary elastics for intermaxillary fixation for four weeks. Upon removing brackets and elastics at four weeks, physical therapy exercises began for one month	No complications were reported. At the one-month follow-up exam, the facial midline was coincident, facial symmetry was achieved, archwires were removed, and intermaxillary elastics were stopped. At the two-month follow-up exam, brackets were removed, and “clinical recovery” was noted. At the 49-month follow-up exam, the patient had normal jaw function devoid of pain
Chan and Au-Yeung [32]	2	M/7 years	Dentate, mixed	Fall from scooter	Bloody otorrhea, inability to verbally communicate	Bilateral condylar fractures, bilateral external auditory canal fractures	Nonsurgical treatment. Soft diet and physical therapy to encourage early jaw mobilization	No follow-up data reported
Kim et al. [33]	3	M/4 years	Dentate, primary	Not reported	Not reported	Bilateral condylar fractures with left mandibular ramus fracture	Closed reduction of right condyle, open reduction with internal semirigid fixation of left condyle	Osteomyelitis of the right mandible developed, necessitating condylectomy. Complications resulted in retrognathic mandible, facial asymmetry, transverse cant of maxillary occlusal plane, and midline discrepancies
Lee et al. [34]	3	M/51 years	Dentate, permanent	Not reported	Not reported	Bilateral condylar fractures, mandibular symphysis fracture, maxillary comminuted fracture, left orbital and nasal bone fractures	Open reduction and internal fixation of the mandibular symphysis and maxillary fracture. Nonsurgical stabilization splint therapy for three months to treat condylar fractures. Additional, comprehensive orthodontic and prosthodontic treatment is provided to treat anterior open bite and occlusal instability	Temporomandibular joint symptoms resolved, and the functional range of mouth opening was recovered following surgery. However, anteroposterior discrepancy, anterior open bite, and occlusal instability remained prior to interdisciplinary comprehensive care. Following comprehensive orthodontic and prosthodontic treatment, stable mandibular position and masticatory function obtained
Bedoya-Rodriguez and Ramirez-Yanez [35]	2	F/11 years	Dentate, mixed	Fall	Limited mouth opening (20 mm), pain and limited movement in lateral excursive movements, pain of TMJs to palpation	Bilateral condylar fracture with medial displacement of condyles	Nonsurgical, conservative functional treatment using a Bionator appliance (ODL Orthodontic Lab, Buffalo, NY) for six months, then an Indirect Planas Tracks (OHI-S, Tallinn, Estonia) appliance for 28 months, plus myofunctional exercises to remodel fracture sites	After 28 months, successful remodeling of condyles was observed with no limitations in mandibular movements. Condylar remodeling was asymmetric and resulted in bifid/heart-shaped condyles. The bifid condylar shape was more pronounced on the left side
Hwang and Ma [36]	3	M/67 years	Edentulous	Fall	Pain and swelling in the left angular region. Limited mouth opening (28 mm)	Comminuted mandibular angle fracture and bilateral condylar fractures	Reduction and fixation of comminuted angle fracture. No surgical treatment of condylar fractures. A monoblock Gunning splint was fabricated and applied with intermaxillary fixation for four weeks	No reported surgical complications. At 42-day post-intermaxillary fixation, mouth opening was measured to be 30 mm, and improved alignment of the condyles was observed
Yamamoto et al. [37]	2	M/58 years	Dentate, permanent	Convulsion	Not reported	Bilateral condylar head fractures with minimally deviated bone fragments	Nonsurgical treatment. No physical therapy rehabilitation was reported	No symptoms or functional deficits were reported
Wu et al. [38]	2	F/6 years	Dentate, mixed	Fall from bicycle	Limited mouth opening (10 mm), pain in the region of the chin	Bilateral condylar fracture	Nonsurgical treatment. Intermaxillary fixation with an occlusal stop for 56 days	No reported complications and improvement in the inclination of the fractured condyle after 56 days of intermaxillary fixation
Sneha et al. [39]	3	M/27 years	Dentate, permanent	Motor vehicle accident	Bilateral preauricular swelling, trismus, anterior open bite	Bilateral condylar fracture, oblique impacted fracture of the symphysis with a displaced inferior border of the mandible and left lingual cortical plate	Mobilization and fixation of impacted mandibular fragments. Reduction and fixation of oblique lingual fracture. No surgical treatment of condyle fractures. Maxillomandibular fixation for two weeks, followed by physical therapy	Pain during mouth opening at three weeks postoperation that reduced with physical therapy. Stable occlusion at six-month follow-up examination
Yoshii et al. [40]	3	M/27 years	Dentate, permanent	Resorption	Bilateral mandibular pain and swelling	Bilateral condyle fracture	-	No reported complications
Bilateral parasymphyseal fractures								
Uppal et al. [41]	2	F/12 years	Dentate, mixed	Initial fractures caused by a motor vehicle accident	Bilateral occlusal step in parasymphysis, constant pain/discomfort, inability to masticate efficiently, mobility of anterior mandibular fragment	Improper reduction and fixation of bilateral parasymphyseal fractures	Plates and screws removed, fragments osteotomized and mobilized, intermaxillary fixation applied with arch bars, and reduction and fixation of parasymphyseal fractures	No complications were reported following the correction of the initial surgical procedure. The patient reported to have “recovered normal oral function and occlusion”
Mulinari-Santos et al. [42]	2	F/10 years	Dentate, mixed	Bicycle accident	Difficulty breathing, posteriorly displaced chin, and steps along inferior borders of mandible	Bilateral parasymphyseal fractures of the mandible with posterior displacement of segment resulting in an obstructed airway	Reduction and fixation of fractures	No surgical complications were reported. At the 21-day postoperative exam, “satisfactory healing” reported
Aires et al. [43]	2	F/19 years	Dentate, permanent	Motorcycle accident	Severe pain in the parasymphyseal region	Bilateral parasymphyseal fracture of the mandible with airway obstruction	Open reduction and internal fixation of fractures	No reported complications
Moafi et al. [44]	2	M/58 years	Edentulous	Physical abuse	Pain in the parasymphyseal region	Bilateral parasymphyseal fracture (right fracture displaced, left fracture complex)	Open reduction and internal fixation of fractures	No surgical complications were reported, and an “uneventful” follow-up appointment noted
Chitlange and Fating [45]	2	M/30 years	Dentate, permanent	Bicycle accident	Pain in parasymphyseal region	Bilateral parasymphyseal fracture of mandible	Open reduction and internal fixation of fractures	No reported complications
Bilateral coronoid process fractures								
Philip et al. [46]	2	F/30 years	Dentate, permanent	Acute reflex contraction of temporalis muscles during the assault	Pain affecting the left side of the face, limited mouth opening (5 mm)	Nondisplaced bilateral transverse coronoid process fractures	No surgical treatment was provided. Conservative management via physical therapy	“Complete resolution of symptoms” by six-week postinjury
Al-Khalisi et al. [47]	2	F/36 years	Dentate, permanent	Unknown contributing factors may include acute reflex contraction of temporalis muscles leading to bilateral stress fractures	Facial pain associated with migraine-like headaches, otalgia, and malocclusion	Bilateral subacute coronoid process fractures without significant displacement and left zygomatic arch linear nondisplaced fractures	Preoperative splint fabrication and use followed by open reduction and internal fixation of fractures, left temporomandibular joint manipulation, and therapy using training elastic for six weeks	No reported complications
Bachaoui et al. [48]	2	F/65 years	Edentulous maxilla, partially edentulous mandible	Unknown contributing factors may include osteoporosis, bruxism, and altered kinetics of temporalis muscle following BSSO (5 years before)	Bilateral discomfort in the TMJ region during function	Bilateral coronoid process fractures with displaced fragments	Surgical removal of displaced coronoid fragments	No reported complications. At the one-week follow-up exam, “a favorable clinical and radiological evolution” was observed
Bilateral angle fractures								
Elavenil et al. [49]	2	M/29 years	Dentate, permanent	Blunt trauma	Inability to close mouth and chew properly	Bilateral mandibular angle fracture	Reduction and fixation of fractures	Not reported
Goyal and Mohanti [50]	2	F/55 years	Dentate, permanent	Fracture secondary to osteoradionecrosis of the mandible	Sudden onset oral pain and inability to open mouth and eat	Bilateral comminuted fractures of the mandibular angles with radiographically visible osteoradionecrosis of the mandible	Reduction and fixation of fractures	Not reported
Reece et al. [51]	2	M/19 years	Dentate, permanent	Fall	Pain along the mandible, malocclusion, facial swelling, trismus	Bilateral mandibular angle fracture	Open reduction and internal fixation of fractures	Not reported
Akmal et al. [52]	2	M/50 years	Dentate, permanent	Fall from bicycle	Pain along the mandible, facial swelling	Bilateral mandibular angle fracture	Open reduction and internal fixation of fractures	Not reported
Bilateral body fractures								
Seshul et al. [53] (case 1)	2	M/65 years	Edentulous	Blunt trauma	Acute respiratory distress due to displaced fracture fragments and prolapse of the tongue into the hypopharynx	Bilateral mandibular body fractures	Reduction and fixation of fractures	No surgical complications were reported. Airway obstruction caused by posterior displacement of the midsection of the mandible was relieved following surgery. No follow-up information reported
Seshul et al. [53] (case 2)	2	M/60 years	Edentulous	Assault	Acute respiratory distress due to displaced fracture fragments and prolapse of the tongue into the hypopharynx	Bilateral mandibular body fractures	Reduction and fixation of fractures	No surgical complications were reported. Airway obstruction caused by posterior displacement of the midsection of the mandible was relieved following surgery. No follow-up information reported
Laskin [54]	2	F/57 years	Partially edentulous	Endosseous dental implant placement	Bilateral pain and swelling	Bilateral mandibular body fractures adjacent to endosseous dental implants that were placed in edentulous posterior mandibular regions	No surgical treatment of fractures was provided. Treatment included an antibiotic prescription and a soft diet	One implant was lost after seven months. Fixed prostheses for the three remaining implants were fabricated, and the patient was reported to have a normal stable occlusion at a three-year follow-up exam
Sidramesh et al. [55]	2	F/68 years	Edentulous	Sudden onset	Inability to close mouth	Bilateral mandibular body	No treatment	No reported complications
Thor [56]	2	M/57 years	Partially edentulous maxilla and mandible	Horse kick to mandible	Displaced anterior mandible with clinical open bite	Bilateral mandibular body fractures	Primary surgery: open reduction and internal fixation using mandibular plates without intermaxillary fixation. Secondary surgery: failing osteosynthesis plates were removed, necrotic tissue was removed, the patient was placed in intermaxillary fixation, a patient-specific implant reconstruction plate was designed and used to reduce fractures, iliac-crest bone graft was harvested and placed into bilateral mandibular defects, oral fistula was closed, and intermaxillary fixation was removed	At a two-week follow-up examination after primary surgery, intraoral fistulas were noted at fracture sites, compromised plate synthesis, and an opening of the bite was observed. No reported complications associated with secondary surgery. At the 10-month follow-up examination after secondary surgery, healing was complete, and “good occlusion” was observed
Florentino et al. [57]	2	M/59 years	Edentulous	Motor vehicle accident	Not reported	Bilateral mandibular body fractures, mandibular atrophy	Reduction and internal fixation of fractures	No surgical complications were reported. “No complaints” noted at 45-day follow-up exam
Ramos et al. [58]	2	M/25 years	Dentate	Physical assault	Inability to chew and pain	Bilateral mandibular body	Open reduction internal fixation	No reported complications
Sindel et al. [59]	2	M/77 years	Edentulous	Fall	Pain, limited mouth opening, bilateral swelling, bilateral paresthesia of lower lip and chin	Bilateral comminuted displaced mandibular body fractures, mandibular atrophy	Open reduction and internal fixation of fractures	No surgical complications were reported. Uneventful healing was observed “during the postoperative period,” but bilateral paresthesia of the lip and chin did not resolve
Hatwar et al. [60]	2	M/20 years	Dentate	Fall	Pain with eating and swelling	Bilateral mandibular body	Open reduction internal fixation	No reported complications
Rahpeyma and Khajehahmadi [61]	2	F/51 years	Dentate	Nerve surgery	Malocclusion	Bilateral mandibular body	Open reduction internal fixation	No reported complications
Asymmetric fractures								
Hemmings [62]	2	M/21 years	Dentate, permanent	Motor vehicle accident	Pain on the left side of neck and face, swelling over left angle and right body of mandible, intraoral step deformity between mandibular molars, limited mouth opening	Right mandibular body fracture and left mandibular angle fracture. Fracture through the body of the second cervical vertebra	Cervical spine fracture immediately immobilized by application of Blackburn caliper. On the third day after injury (following reduction in swelling), mandibular fractures were reduced and immobilized via upper/lower arch bars, and intermaxillary fixation was applied using elastic bands for five weeks. The patient nursed on a rotatable Stryker bed for six weeks, after which chest plaster and halo fixation were applied for one month, and then a neck collar was applied for an additional month	Not reported
Sindet-Pedersen et al. [63]	2	M/28 years	Dentate, permanent	Assault	Bilateral swelling of the submandibular region, reduced mobility of the mandible.	Right mandibular angle fracture and left mandibular body fracture	The patient initially refused treatment. Several hours later, the patient returned to the hospital with a large hematoma in the right tonsillar region that extended past the midline (the patient had hemophilia). The fracture is provisionally immobilized with a stainless-steel ligature tie. Hematomas treated with activated PCC (5,000 unit doses twice daily for six days). After six days, activated PCC continued, and oral tranexamic acid (2 g four times per day) and mouthwash (10 mL 5% tranexamic acid solution) started for one day. At day 7, brackets bonded to facial tooth surfaces and elastics applied for maxillomandibular fixation and oral rinses with 5% tranexamic acid were continued for two weeks	Four weeks following treatment, the patient experienced bleeding from the right third molar region, and 5% tranexamic acid mouth rinse was reinstated. Six weeks after treatment, elastics were removed, and occlusion was reported to be normal
Wright et al. [64]	2	F/40 years	Edentulous	Fall	Right-sided facial swelling, right-sided mental nerve and facial nerve paresthesia, right facial musculature paralysis	Right displaced mandibular body fracture and left displaced condylar neck fracture	Mandibular body fracture treated with Gunning splints. Right facial muscles treated with a six-week course of galvanic stimulation	No surgical complications were reported. Following the six weeks of galvanic stimulation, at a six-month postoperative examination, facial paralysis was confined to the lower lip, and bony healing was observed. At the nine-month postoperative examination, full function of facial muscles was restored
Brookes [65]	2	F/72 years	Partially edentulous maxilla and mandible	Osteolytic bone lesions secondary to chronic lymphocytic leukemia	Pain and swelling in left mandible during eating (left body fracture), two weeks later, development of an anterior open bite (right angle fracture)	Left mandibular body fracture, then a subsequent right mandibular angle fracture with multiple osteolytic bone lesions throughout facial bones (and throughout bodily skeleton)	Extraction of teeth in hyperocclusion, treatment with pamidronate (bisphosphonate inhibitor), and radiotherapy to the mandible	Pathological fractures continued with fractures of the right radius, left shaft of the humerus, and right shaft of the femur. Mandibular fractures never resolved, and the patient passed away 12 months following mandibular fractures
Crean et al. [66]	2	F/2 years	Dentate, primary	Horse kick to the mandible	Small facial laceration, bruising, sublingual hematoma	Right parasymphyseal fracture, left angle fracture	No surgical treatment was provided. A soft diet for three weeks and a suggestion to avoid physical contact with the jaw	Complete recovery with “maintenance of good occlusion and stabilization of the fractures” seen at three months postinjury follow-up examination
Guven et al. [67]	2	M/11 years	Dentate	Car accident	Difficulty opening and deviation of the mandible	Mandibular body and condyle	Open reduction internal fixation	No reported complications
Smith et al. [68]	3	M/74 years	Dentate, permanent	Fall, the patient has Charcot-Marie-Tooth disease and was predisposed to frequent falls	Malocclusion with a traumatic step between mandibular left canine and lateral incisor. Limited mouth opening (20 mm). Pain and mandible deviation to the right upon opening	Displaced right condylar fracture, displaced left parasymphyseal fracture, nondisplaced left mandibular ramus fracture	Manual reduction of parasymphyseal fracture, then the patient was placed in intermaxillary fixation. The parasymphyseal fracture was fixed, and the condylar fracture was reduced and fixed before the removal of intermaxillary fixation. Light elastic traction was used to guide the patient into occlusion for two weeks, and physical therapy was performed	At the six-week postoperative exam, the radiograph displayed a good bony reduction of fracture segments, the patient’s interincisal opening was 40 mm, and the patient had returned to his normal diet
Wang et al. [69]	3	M/58 years	Edentulous maxilla, partially edentulous mandible	Motor vehicle accident	Decreased sensation in the distribution of mental nerves bilaterally	Right mandibular body fracture, left mandibular angle fracture, and left intracapsular condylar fracture	Intermaxillary fixation using a custom maxillomandibular splint, reduction of right body and left angle fractures	No reported surgical complications. At the eight-week follow-up exam, the patient reported residual numbness of bilateral lower lips
Dal Santo [70]	2	M/10 years	Dentate	Motor vehicle accident 16 years before	None reported	Right mandibular body, left angle	Open reduction internal fixation	No reported complication

Among the reported cases that involved bilateral mandibular fractures with an associated symphysis fracture (three fracture sites), the vast majority reported bilateral condylar/subcondylar fractures with the symphysis fracture (Table 2). Management strategies varied considerably across cases. In younger patients, many of the condylar fractures were managed with nonsurgical, conservative treatment using functional appliances or intermaxillary fixation [35,57,64]. In older patients, surgical management of the condylar fractures was more common (Table 2). For most reported cases, both surgical management of adult condylar fractures and nonsurgical management of adult and childhood fractures resulted in improvements in condylar inclination and successful condylar remodeling. Nevertheless, three cases were reported of patients with condylar ankylosis several years following condylar fracture. Additionally, surgical treatment of bilateral condylar fractures in a pediatric patient (age of four years) resulted in osteomyelitis of the right condyle that necessitated condylectomy and significantly impaired growth of the mandible [33].

Several bilateral mandibular body fractures without a concomitant symphysis fracture have been reported. Among these, one case was caused by a horse kick to a partially edentulous mandible, one case involved a fall with a completely edentulous and atrophic mandible, two cases were caused by blunt trauma/assault to edentulous mandibles, one case occurred during a motor vehicle accident, and one case was caused by improper placement of endosseous dental implants into a partially edentulous mandible [33,53,54,56,57,59]. Notably, unlike our presented case in which bilateral mandibular body fractures occurred in a completely dentate mandible, these six reported cases all involved completely edentulous mandibles (four of six cases) or partially edentulous mandibles missing nearly all posterior teeth (two of six cases). Above all, previous studies have shown a low incidence of mandible fractures induced by gunshot wounds [71].

Bilateral mandibular body fractures are known to potentially cause acute respiratory distress as the midsection of the mandible can be displaced posterior-inferiorly into the hypopharynx due to an unopposed pull from the soft tissues of the tongue and floor of the mouth [53]. This complication was not observed in our case, potentially due to the concomitant symphysis fracture serving to redistribute forces exerted by soft tissues. In general, fractures of the mandibular bodies, mandibular angles, and parasymphyseal regions were successfully treated with reduction/fixation alongside intermaxillary fixation. Notable exceptions include an individual with multiple osteolytic bone lesions secondary to chronic lymphocytic leukemia who experienced extensive pathologic fractures of the facial bones and bodily skeleton [64]. Additionally, three cases reported remnant paresthesia in the distribution of the mental nerves following mandibular body fractures, even with successful reduction and fixation of the fractures [59,65,69]. As highlighted by a recent review, significant uncertainty still exists regarding how to best manage mandibular fractures [72]. In the presented case, reduction and fixation of the fracture sites with three small fixation plates and one large reconstruction plate resulted in a stable and repeatable occlusion at six weeks (Figure 4), with the patient reporting no dysfunction nor signs of paresthesia. Overall, the transcervical approach provides a great approach to reducing comminuted fractures with minimal complications [73].

## Conclusions

In conclusion, to our knowledge, this is the first reported case of a mandibular symphysis fracture with concomitant bilateral mandibular body fractures. The gunshot wound to the parasymphyseal region may have predisposed the mandible to this unique fracture pattern. Surgical reduction and fixation of the fracture sites resulted in a stable and successful outcome. Knowledge of the mechanism-based factors resulting in different patterns of mandibular injury is valuable to the surgeon in the management of mandibular fractures. As such, this case provides valuable insight to enhance our understanding of such mechanism-fracture pattern correlations.
